# Sex-related differences in left ventricular remodeling and outcome after alcohol septal ablation in hypertrophic obstructive cardiomyopathy: insights from cardiovascular magnetic resonance imaging

**DOI:** 10.1186/s13293-022-00447-x

**Published:** 2022-07-07

**Authors:** You-Zhou Chen, Xing-Shan Zhao, Jian-Song Yuan, Yan Zhang, Wei Liu, Shu-Bin Qiao

**Affiliations:** 1 Department of Cardiology, Beijing Jishuitan Hosptial, No. 31 East Street, Xinjiekou, XiCheng, Beijing, 100035 China; 2 Department of Cardiology, National Center for Cardiovascular Disease, Fuwai Hospital, Chinese Academy of Medical Sciences and Peking Union Medical College, 167 Beilishi Road, XiCheng, Beijing, 100037 China; 3 Department of Magnetic Resonance Imaging, National Center for Cardiovascular Disease, Fuwai Hospital, Chinese Academy of Medical Sciences and Peking Union Medical College, 167 Beilishi Road, XiCheng, Beijing, 100037 China

**Keywords:** Hypertrophic cardiomyopathy, Alcohol septal ablation, Remodeling, Sex

## Abstract

**Background:**

Alcohol septal ablation (ASA) has been proven to reverse left ventricular (LV) remodeling in hypertrophic cardiomyopathy (HCM). However, there are no studies on the effect of sex on LV remodeling after ASA. We aimed to investigate whether sex differences affect the process of LV remodeling and outcome after ASA.

**Methods:**

A total of 107 patients with obstructive HCM (54 men and 53 women, mean age 51 ± 8 years) were recruited. Cardiovascular magnetic resonance (CMR) was performed at baseline and 16 months after ASA. The extent of late gadolinium enhancement (LGE) was measured.

**Results:**

Women had a higher indexed LV mass and smaller indexed LV end-systolic volumes than men at the time of ASA. After ASA, both men and women exhibited a regression of LV mass, and the percentage of mass regression was greater in men than women (15.3% ± 4.3% vs. 10.7% ± 1.8%, *p* < 0.001). In multivariable analysis, male sex, higher reduction of LV outflow tract (LVOT) gradient and lower baseline LV mass index were independently associated with greater LV mass regression after ASA. Kaplan–Meier analysis showed significantly higher cardiovascular events in women than in men (p = 0.015). Female sex [hazard ratio (HR) 3.913, *p* = 0.038] and LV mass preablation (HR, 1.019, *p* = 0.010) were independent predictors of cardiovascular outcomes.

**Conclusions:**

Males with HCM had favorable reverse remodeling with greater LV mass regression post-ASA than female patients. This favorable LV reverse remodeling might provide a mechanistic explanation for the survival advantage in men.

## Highlights

1. Female patients with HCM showed worse LV remodeling with a higher indexed LV mass and a smaller indexed LV end-diastolic volume (measured by CMR) at the time of ASA.
2. Both men and women exhibited the LV reverse remodeling, however, men experienced more favorable LV reverse remodeling than women after ASA. The overall percentage of the LVM index regression was greater among men than women.
3. Women with HCM had worse relative composite endpoint than men. Sex and LV mass preablation were independent predictors of cardiovascular outcomes.
4. Sex appears to be a significant modifier in HCM patients receiving ASA treatment and highlighted the need for a different approach to women with HCM, such as improving women’s awareness of diagnosis and follow-up management as well as earlier referral for advanced therapies (e.g., septal reduction therapy and ICD implantation).

## Background

Hypertrophic cardiomyopathy (HCM) is the most prevalent monogenetically inherited cardiac disease and is characterized by unexplained left ventricular (LV) hypertrophy, myocardial fibrosis and dynamic LV outflow tract (LVOT) obstruction [1]. Sex-related differences have been observed in the phenotypic expression and outcomes in HCM patients, and women are prone to have a higher prevalence of the obstructive phenotype and more commonly develop advanced heart failure during follow-up [2–5]. However, there is some uncertainty as to whether women with HCM have a greater degree of ventricular remodeling than men.

Alcohol septal ablation (ASA), as an alternative to myectomy, has been shown to reduce LVOT obstruction and lead to significant improvement in both symptoms and functional capacity. Previous studies have demonstrated that ASA is associated with reductions in symptoms and regression of LV remodeling [6–8]. However, there is a paucity of data describing sex-related differences in LV remodeling and outcome after ASA. Cardiovascular magnetic resonance (CMR) imaging can provide detailed information regarding LV hypertrophy, ventricular function and the presence and extent of myocardial fibrosis. The aim of our study was to evaluate sex differences in the changes and potential modulating factors of LV remodeling as well as the clinical outcome in the long-term follow-up after ASA.

## Methods

### Patients

The patients were enrolled from Fuwai Hospital. All patients gave informed consent to participate in the study. The study was conducted in accordance with the Fuwai Hospital Ethics Committee’s guidelines. We retrospectively studied 107 patients (54 men, 53 women) with obstructive HCM who received ASA procedures from January 2009 to December 2013. The indications for ASA are obstructive HCM with severe drug refractoriness, New York Heart Association (NYHA) class III/IV symptoms and a resting LVOT gradient of ≥ 40 mmHg or > 100 mmHg during provocation [9]. There were no patients with previous surgical myectomy, aortic valvular disease, coronary artery disease or general contraindications to CMR [9]. Doppler echocardiography and color flow imaging were used to ascertain the peak LVOT gradient and mitral regurgitation (MR) (grade I, mild; grade II, moderate; grade III, moderate to severe; grade IV, severe) [10].

### ASA procedure

Septal ablation was performed in 107 patients with previously described methods [11]. In brief, through a 7F left coronary guiding catheter, an oversized, over-the-wire angioplasty balloon was placed in the septal perforator artery. Angiographic contrast was injected through the balloon catheter to identify the perfusion bed of the septal perforator. After delineation of the targeted myocardium with contrast, 1 to 3 ml of ethanol was slowly injected. A successful procedure was defined by a reduction in the LVOT pressure gradient of > 50% of the baseline value.

### CMR imaging and data analysis

CMR imaging studies were performed in 107 patients before ASA and after ASA in the follow-up. All scans were performed using a 1.5-T clinical scanner (Siemens Medical Solutions, Erlangen, Germany). To evaluate functional parameters, electrocardiographic gating cine images were acquired using a segmented, balanced, steady-state free-precession sequence. After scout images, cine images were obtained in four-chamber, three-chamber and two-chamber long- and short-axis views that covered the entire LV from base to apex. Contrast-enhanced late gadolinium enhancement (LGE) images were acquired 10 min after intravenous administration of 0.2 mmol/kg gadolinium dehydrate using a segmented phase-sensitive inversion-recovery spoiled gradient-echo sequence.

All CMR image analyses were performed using commercial software (Medis Medical Imaging systems, Netherlands) by single experienced radiologic technicians blinded to the clinical and procedural data. Epicardial and endocardial borders of the LV myocardium at end-diastole and end-systole with papillary muscles and trabeculations excluded before and after ASA were manually traced during the whole cardiac phase to obtain LV end-diastolic and end-systolic volumes, ejection fractions and myocardial mass. The LV mass (LVM) index, end-diastolic volume (EDV) index and end-systolic volume (ESV) index were indexed to body surface area (BSA). After contrast-enhanced DHE images were acquired, the small patchy areas of hyperenhancement were clearly noted in the IVS outside the infarct region. For analysis of the LGE images, each slice was visually inspected by two experienced independent observers who were blinded to the clinical and procedural data.

Infarct size after ASA was evaluated by manual tracing of the hyperenhanced area, and these small patchy hyperenhanced areas within the septal myocardium were determined with pixel SI values > 4 SD of remote nonenhanced myocardium as the postablation infarct [12]. The center of the infarct area was defined as the center of the hyperenhanced areas within the septal myocardium on the short-axis images with the largest area of hyperenhancement. Central dark zones within the area of hyperenhancement were included. The summation of the planimetered LGE areas in all short-axis slices yielded the total LGE extent (including the infarct size). Other remote hyperenhancement areas away from the areas at the alcohol ablation position within the septal myocardium were defined as remote LGE [8]. Maximal septal and later wall thickness were measured at end-diastole on the mid-ventricular short-axis cine using electronic caliper measurement tools.

### Follow-up and outcomes

A composite cardiovascular endpoint was defined as the primary outcome, which consisted of new-onset atrial fibrillation, sustained ventricular tachycardia, new-onset or acute chronic heart failure (defined as deterioration of New York Heart Association functional class III or IV) requiring hospitalization, and cardiovascular death.

### Statistical analysis

Data are presented as the mean ± SD for normally distributed continuous variables and as medians and interquartile ranges for non-normally distributed continuous variables. Differences between means were evaluated using paired and unpaired (for independent group comparisons) Student’s *t* tests for normally distributed data and the Mann–Whitney or Wilcoxon signed rank test for nonparametric data. The *χ*^2^ test was employed to compare categories of data. Pearson correlation coefficients were used to investigate the relationships between the changes in parameters. Predictors of the ratio of LV mass reduction were calculated using a stepwise multiple linear regression model with baseline measurements entered as covariate factors. Variables with a *p* < 0.05 were entered into the multivariable analysis. The composite endpoint was plotted according to the Kaplan–Meier method, and was compared using the log-rank test. Univariate and then multivariate analyses to determine the relative contribution of variables to overall mortality were examined by the Cox regression model. Statistical significance was defined as *p* < 0.05. All statistical analyses were performed using SPSS version 18.0 for Windows (SPSS, Inc., Chicago, Illinois).

## Results

### Clinical and demographic data

A total of 107 patients, including 54 men and 53 women, were recruited for the study. The baseline clinical characteristics of the patients are described in Table 1. The mean age of the recruited patients was 51 ± 8 years (47 ± 8 years in men vs. 55 ± 6 years in women, *p* < 0.001). Women had a higher prevalence of dyspnea and a higher NYHA class than men (*p* < 0.05). There were no significant differences in the prevalence of hypertension, diabetes, atrial fibrillation, nonsustained ventricular tachycardia or medications between sexes. Women had more severe mitral regurgitation (*p* < 0.05). Regarding treatment, there were no differences in the use of beta receptor antagonists, calcium channel blockers, angiotensin-converting enzyme inhibitors or angiotensin receptor blockers.

**Table 1:** Baseline clinical and echocardiographic characteristics

	Men (*n* = 54)	Women (*n* = 53)	*p* Value
Age (year)	47 ± 8	55 ± 6	< 0.001
BSA (m^2^)	1.9 ± 0.1	1.8 ± 0.1	< 0.001
HR (bpm)	77 ± 9	74 ± 9	0.338
SBP (mm Hg)	129 ± 12	132 ± 17	0.382
DBP (mm Hg)	73 ± 12	75 ± 13	0.342
Menopausal status	NA	48 (90.6%)	
NYHA (median)	2 (1, 4)	3 (2, 4)	< 0.001
Hypertension	3 (5.6%)	4 (7.5%)	0.677
Diabetes	3 (5.6%)	2 (3.8%)	0.662
Nonsustained VT	5 (9.3%)	6 (11.3%)	0.974
Chest pain	11 (20.4%)	20 (37.7%)	0.077
Dyspnea	27 (50.0%)	39 (73.6%)	0.021
Syncope	4 (7.4%)	3 (5.7%)	0.715
Family history of HCM or sudden death	7 (12.9%)	5 (9.4%)	0.786
Medications			
Beta blockers	32 (59.3%)	27 (50.9%)	0.503
Nondihydropyridine CCB	8 (14.8%)	15 (28.3%)	0.144
ACEI/ARB	8 (14.8%)	13 (24.5%)	0.307
Diuretics	0 (0.0%)	3 (5.7%)	0.235
Moderate or severe MR	28 (51.9%)	39 (73.6%)	0.037
Alcohol use	2.3 ± 0.5	1.6 ± 0.4	< 0.001

Data are expressed as the mean ± SD, number (percentage), or median (interquartile range)

*BSA*: body surface area, *SBP* systolic blood pressure, *DBP* diastolic blood pressure, *NYHA* New York Heart Association; *VT* ventricular tachycardia; *SCD* sudden cardiac death; *HCM* hypertrophic cardiomyopathy, *CCB* calcium channel blocker, *ACEI* angiotensin-converting enzyme inhibitor, *ARB* angiotensin receptor blocker, *MR* mitral regurgitation, *LVOTG* left ventricular outflow tract gradient

### Baseline CMR left ventricular measurements

At baseline before ASA, women with obstructive HCM had a higher indexed LV mass than men (108.6 ± 16.6 vs. 95.1 ± 15.6 g/m^2^, *p* = 0.001) and smaller indexed LV end-systolic volumes (21.8 ± 2.9 vs. 24.8 ± 2.9 ml/m^2^, *p* = 0.006) and higher ejection fraction (67.2% ± 4.4% vs. 64.1% ± 3.5%). There were no significant differences in indexed LV end-diastolic volume (*p* = 0.129) or septal thickness (*p* = 0.419) between men and women. Remote LGE was observed in 90.1% of patients. The incidence of LGE was not significantly different between women and men (94.4% vs. 85.7%, *p* = 0.217). However, women showed more LGE (12.2 ± 2.3 vs. 9.9 ± 2.9 g, *p* = 0.001) and a greater LVOT gradient (100.1 ± 18.9 vs. 90.6 ± 10.9 mmHg, *p* = 0.012) than men. LGE was significantly related to the LVM index not only in men (*r* = 0.564, *p* = 0.001), but also in women (*r* = 0.489, *p* = 0.003) (Table 2).

**Table 2:** Cardiac magnetic resonance data before and after ASA according to sex

	Men (*n* = 54)	Women (*n* = 53)	*p* value for sex difference
Septal thickness (mm)			
Baseline	21.0 ± 1.8	21.5 ± 2.8	0.419
Follow-up	15.1 ± 1.5	16.5 ± 2.7	0.009
Changes	5.9 ± 1.6	5.0 ± 1.5	0.013
*p* value	< 0.001	< 0.001	
Posterior wall thickness (mm)			
Baseline	10.2 ± 1.4	10.9 ± 1.8	0.046
Follow-up	8.6 ± 1.1	9.8 ± 1.6	< 0.001
Changes	1.6 ± 0.7	1.1 ± 0.7	0.005
*p* value	< 0.001	< 0.001	
LVM index (g/m^2^)			
Baseline	95.1 ± 15.6	108.6 ± 16.6	0.001
Follow-up	80.8 ± 15.7	97.1 ± 15.9	< 0.001
Changes	14.3 ± 4.1	11.4 ± 1.9	0.001
*p* value	< 0.001	< 0.001	
Post-ablation infarct (g)	11.2 ± 2.8	7.8 ± 2.2	< 0.001
LVM reduction ratio, %	15.3 ± 4.3	10.7 ± 1.8	< 0.001
LVEDV index (g/m^2^)			
Baseline	69.5 ± 7.2	66.8 ± 7.6	0.129
Follow-up	77.0 ± 7.6	72.2 ± 6.9	0.006
Changes	7.5 ± 2.3	5.4 ± 1.3	< 0.001
*p* value	< 0.001	< 0.001	
LVESV index (ml/m^2^)			
Baseline	24.8 ± 2.9	21.8 ± 2.9	0.006
Follow-up	26.5 ± 3.3	23.2 ± 2.7	< 0.001
Changes	1.7 ± 0.8	1.4 ± 0.7	0.102
*p* value	< 0.001	< 0.001	
EF (%)			
Baseline	64.1 ± 3.5	67.2 ± 4.4	0.002
Follow-up	65.5 ± 3.6	67.8 ± 3.9	0.014
Changes	1.4 ± 0.04	0.5 ± 0.02	0.015
*p* value	< 0.001	0.006	
Remote LGE (g)			
Baseline	9.9 ± 2.9	12.2 ± 2.3	0.001
Follow-up	9.9 ± 2.9	12.3 ± 2.3	0.001
Changes	0.05 ± 0.19	0.10 ± 0.36	0.433
*p* value	0.153	0.100	
LVOTG (mmHg)			
Baseline	90.6 ± 10.9	100.1 ± 18.9	0.012
Follow-up	15.2 ± 8.4	31.1 ± 9.1	< 0.001
Changes	75.4 ± 9.5	68.9 ± 13.3	0.022
*p* value	< 0.001	< 0.001	

*LV* left ventricular, *LVM* left ventricular mass, *EDV* end-diastolic volume, *ESV* end-systolic volume, *EF* ejection fraction, *LGE* late gadolinium enhancement, *LVOTG* left ventricular outflow tract gradient

### CMR measurements post-ASA in the follow-up

**Fig. 1:**
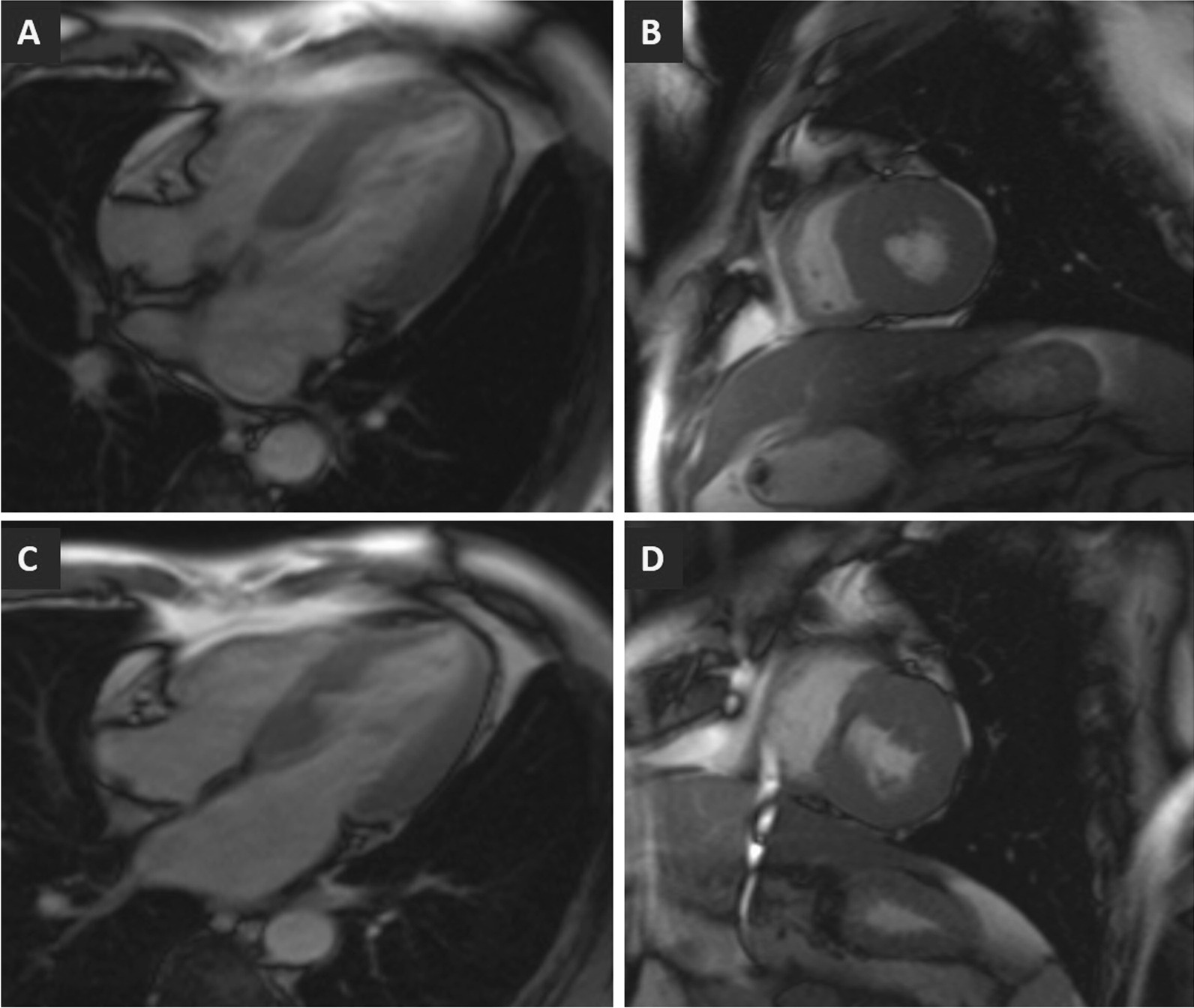
Short-axis and four-chamber CMR images of the LV acquired at the end-diastole. The upper panel (**A** and **B**) depicts the male ventricle with an LVM index of 81.12 g/m^2^ and an LVEDV index of 62.76 ml/m^2^ at baseline. The lower panel (Figure C and D) depicts the reverse remodeling of the ventricle with an LVM index of 70.55 g/m^2^ and a LVEDV index of 69.23 ml/m^2^ in the follow-up. *LV*: left ventricle, *LVM*: left ventricular mass, *LVEDV* left ventricular end-diastolic volume

**Fig. 2:**
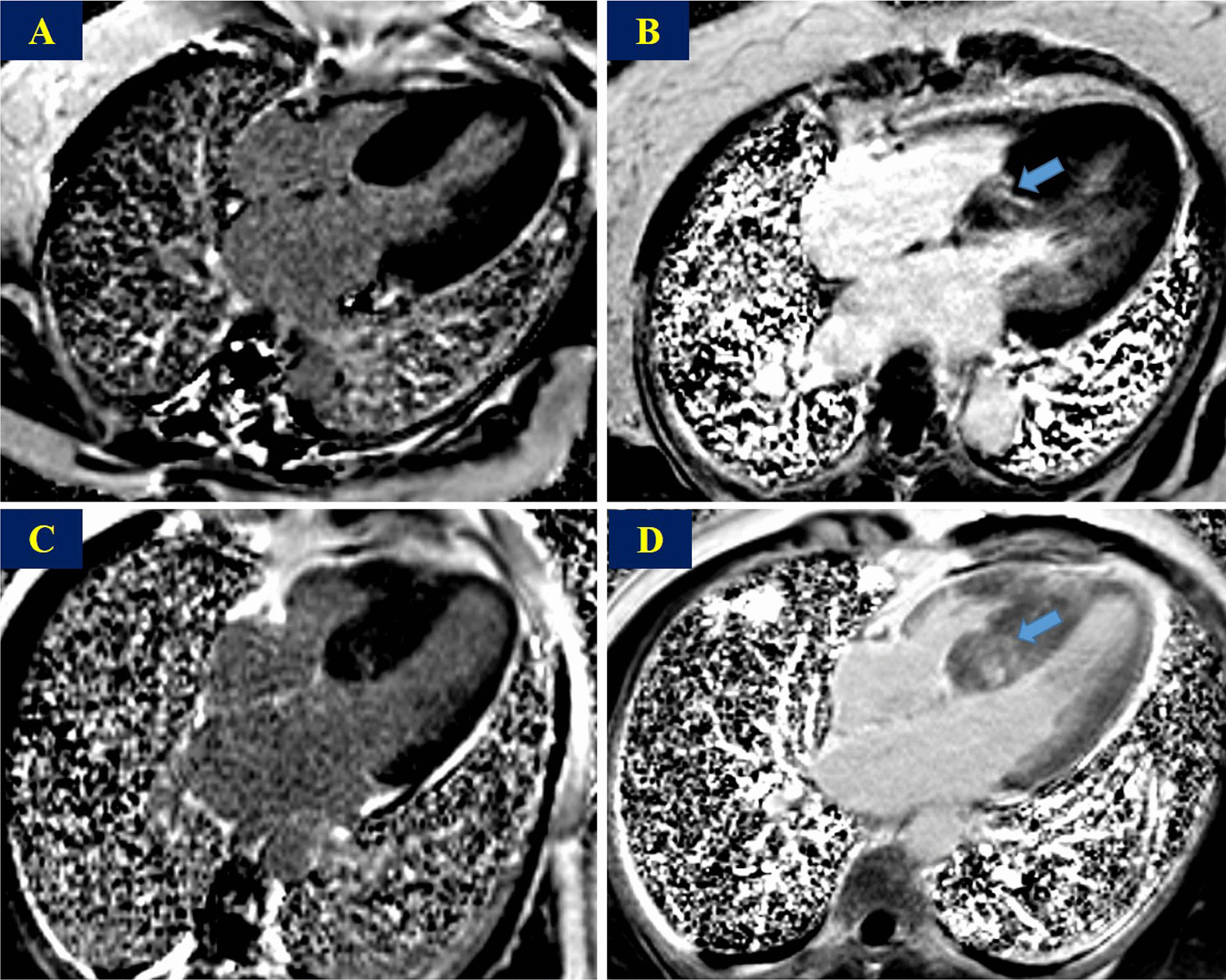
Contrast-enhanced magnetic resonance images in one female patient before ASA with an LVM index of 151.74 g/m^2^, an LVEDV index of 79.61 ml/m^2^ and a remote LGE of 13.52 g (**A**) and at follow-up after ASA with an LVM index of 141.26 g/m^2^, an LVEDV index of 84.61 ml/m^2^ and a remote LGE of 13.47 g (**B**) and in one male patient before ASA with an LVM index of 107.04 g/m^2^, an LVEDV index of 83.84 ml/m^2^ and a remote LGE of 9.16 g (**C**) and at follow-up after ASA with an LVM index of 90.59 g/m^2^, an LVEDV index of 89.09 ml/m^2^ and a remote LGE of 9.09 g (**D**). The blue arrows indicate the position of the alcohol ablation. *ASA* alcohol septal ablation, *LVM* left ventricular mass, *LVEDV* left ventricular end-diastolic volume, *LGE* late gadolinium enhancement

**Fig. 3:**
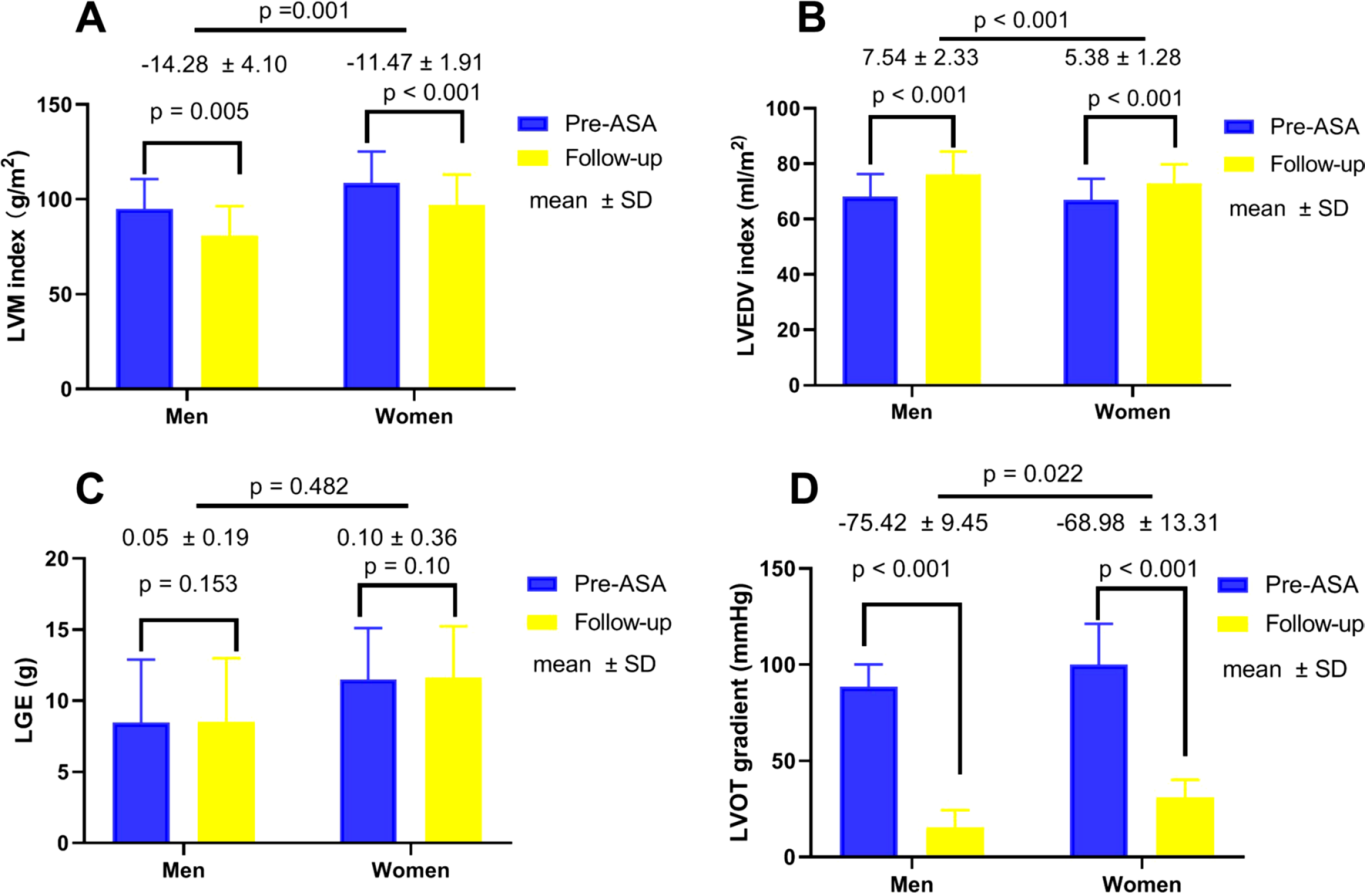
Comparison of **A** LVM index, **B** LV EDV index, **C** LGE and **D** left ventricular outflow tract gradient in men (*n* = 54) versus women (*n* = 53) at baseline and long-term follow-up after ASA *LVM* left ventricular mass, *LVEDV* left ventricular end-diastolic volume, *LGE* late gadolinium enhancement, *ASA* alcohol septal ablation

The reverse remodeling parameters according to sex are shown in Table 2. After ASA, there was a significant reduction in the LVM index and septal thickness in both groups (*p* < 0.001) (Fig. 1). Examples of LV remodeling before and post-ASA in both males and females are shown in Fig. 2. Men experienced greater absolute LV mass regression (14.3 ± 4.1 vs. 11.5 ± 1.9 g/m^2^, *p* = 0.001) than women. Furthermore, when expressed as a percentage reduction of baseline LVM index, the percent of mass regression was still greater in men than women (15.3% ± 4.3% vs. 10.7% ± 1.8%, *p* < 0.001). In addition, there were significant increases in the LVEDV index and LVESV index in both groups (*p* < 0.001). Men had a higher increase in the LVEDV index (7.5 ± 2.3 vs. 5.4 ± 1.3 ml/m^2^, *p* < 0.001) and a similar increase in the LVESV index (1.7 ± 0.8 vs. 1.4 ± 0.7 ml/m^2^, *p* = 0.102) than women. ASA also resulted in significant reductions in the LVOT gradient at rest in both groups (*p* < 0.001). Men had a higher LVOT gradient reduction (75.4 ± 9.5 vs. 68.9 ± 13.3 mmHg, *p* = 0.022) than women. After ASA, there were no significant changes in remote LGE in either group (*p* = 0.482, seen in Fig. 3). The percentage reduction in LV mass was related to the baseline LVM index (*r* = − 0.512, *p* < 0.001), baseline LGE (*r* = − 0.417, *p* < 0.001) and LVOT gradient (*r* = − 0.461, *p* < 0.001) in the whole group. Furthermore, the change in the LVM index was significantly associated with the LVEDV index increment (*r* = − 0.249, *p* = 0.036).

## Predictors of reverse remodeling

Clinical variables, including patient demographics and CMR-measured parameters, were analyzed to determine the predictors of reverse remodeling. In the univariate analysis, age, sex, LVOT gradient, baseline LVM index, postablation infarct size and remote LGE appeared to predict the reduction in LV mass (*p* < 0.001). However, on multivariable analysis, only sex (*p* = 0.007), baseline LVM index (*p* = 0.006) and the reduction of LVOT gradient (*p* < 0.001) were independent predictors for LV mass regression (Table 3). Furthermore, we also performed binary logistic regression analysis to examine the predictors of favorable LV remodeling according to the degree of LV mass reduction. As shown in Table 4, significant LV reverse remodeling (> 12% decrease in baseline LV mass index) was observed in 51% of our cases. The main correlates of significant LV reverse remodeling in the univariate analysis were sex (OR = 0.176, *p* = 0.001), LVOT gradient (OR = 0.958, *p* = 0.048), baseline LVESV index (OR = 1.771, *p* = 0.044) and infarct size (OR = 1.256, *p* = 0.024). In multivariate analysis, sex was the only variable significantly associated with LV reverse remodeling [OR = 0.132 (95% CI 0.038–0.456), *p* = 0.001] (Table 4).

**Table 3:** Predictors of the reduction of LVM after ASA by multivariable logistic regression

	Univariate analysis		Multivariate analysis	
	Beta ± SE	*p* value	Beta ± SE	*p* value
Age	− 0.44 ± 0.001	< 0.001		
Sex	− 0.58 ± 0.008	< 0.001	− 0.31 ± 0.009	0.007
BSA	0.30 ± 0.031	0.011		
HR	− 0.02 ± 0.001	0.902		
SBP	− 0.06 ± 0.00	0.626		
DBP	− 0.08 ± 0.00	0.495		
Menopausal status	− 0.17 ± 0.00	0.319		
Hypertension	− 0.04 ± 0.005	0.741		
Diabetes	− 0.06 ± 0.019	0.624		
Atrial fibrillation	− 0.21 ± 0.013	0.087		
LVOT gradient reduction	− 0.46 ± 0.000	< 0.001	− 0.389 ± 0.000	< 0.001
MR changes	0.08 ± 0.008	0.493		
Baseline septal thickness	− 0.09 ± 0.002	0.443		
Baseline LVM index	− 0.51 ± 0.001	< 0.001	− 0.305 ± 0.000	0.006
Baseline LVEDV index	− 0.002 ± 0.001	0.987		
Baseline LVESV index	− 0.15 ± 0.001	0.223		
Post-ablation infarct size	0.57 ± 0.002	< 0.001		
Remote LGE	− 0.42 ± 0.001	< 0.001		

*BSA* body surface area, *SBP* systolic blood pressure, *DBP* diastolic blood pressure, *MR* mitral regurgitation, *LVOTG* left ventricular outflow tract gradient, *LV* left ventricular, *LVM* left ventricular mass, *EDV* end-diastolic volume, *ESV* end-systolic volume, *LGE* late gadolinium enhancement, *LVOT* left ventricular outflow tract

**Table 4:** Predictors of the reversal of LVM remodeling (decrease in LVM index > 12% of baseline LVM index) after alcohol septal ablation by multivariable logistic regression

	Univariate analysis			Multivariate analysis	
	OR	*p* value	OR	95% *CI*	*p* value
Age	0.974	0.348			
Sex	0.176	0.001	0.132	[0.038, 0.456]	0.001
HR	1.00	0.990			
Menopausal status	0.644	0.586			
Hypertension	1.029	0.906			
Diabetes	0.221	0.188			
Atrial fibrillation	0.604	0.468			
LVOTG reduction	0.958	0.048			
MR changes	1.101	0.799			
Baseline septal thickness	1.132	0.238			
Baseline LVM index	0.985	0.272			
Baseline LVEDV index	1.067	0.056			
Baseline LVESV index	1.171	0.044			
Post-ablation infarct	1.256	0.024			
Remote LGE	0.950	0.367			

*HR* heart rate, *MR* mitral regurgitation, *LVOTG* left ventricular outflow tract gradient, *LV* left ventricular, *LVM* left ventricular mass, *EDV* end-diastolic volume, *ESV* end-systolic volume, *EF* ejection fraction, *LGE* late gadolinium enhancement, *LVOTG* left ventricular outflow tract gradient

### Outcome analysis

**Fig. 4:**
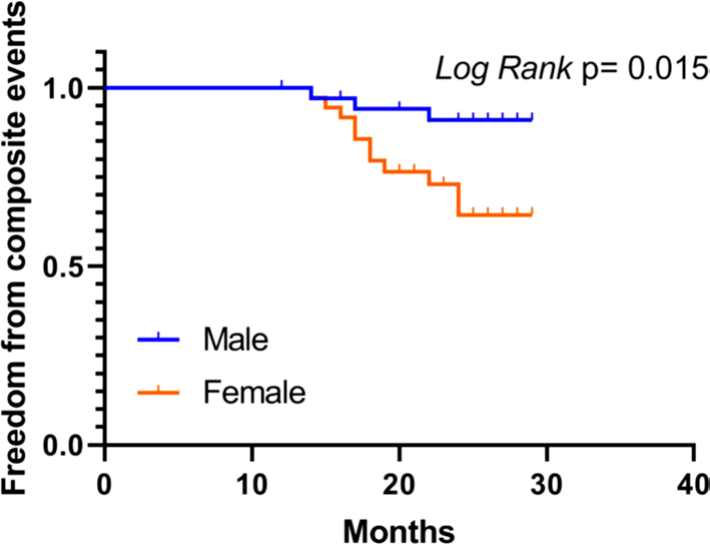
Kaplan–Meier curve of the composite endpoint in female patients undergoing ASA vs. male patients

During a median follow-up period of 25 months (range, 12–29 months), a total of 14 patients (3 men and 11 women) were admitted to the hospital because of new-onset acute heart failure and sustained new-onset atrial fibrillation that reached a cardiovascular endpoint. No patients died in the follow-up. Kaplan–Meier analysis showed significantly worse composite endpoint in women than in men after ASA (log rank = 5.947, *p* = 0.015, Fig. 4). Multivariate analyses showed that independent predictors of cardiovascular events in patients undergoing ASA were female sex (HR, 3.913; 95% CI, 1.080–14.177; *p* = 0.038) and LV mass pre-ASA (HR, 1.019; 95% CI, 1.005–1.034; *p* = 0.010).

## Discussion

This study assessed the influence of sex on differences in LV remodeling and outcome in obstructive HCM after ASA in long-term follow-up using CMR imaging. Our current results demonstrated that both men and women had favorable reverse remodeling with significant LV mass regression and EDV increments in the long-term follow-up. Women experienced more clinical events than men after ASA in the follow-up. The overall percentage of the LVM index regression was greater among men. Additionally, male sex, baseline LVM index and change in the LVOT gradient were independently associated with the LV remodeling process, indicating that these factors could be used as indicators for LV mass regression after ASA in patients with HCM. The greater changes in LV reverse remodeling in males might contribute to the better prognosis after ASA compared to females.

Although population-based studies have demonstrated that sex plays an important role in the prevalence, severity and prognosis of HCM, female sex was associated with decreased survival [2–5]. However, the mechanisms of sex-related differences with respect to prognosis remain unknown. Schulz-Menger et al. found that females with obstructive HCM appeared to experience a significantly larger degree of remodeling, which was mainly determined by relatively increased myocardial mass than male patients [13]. In contrast, Lu et al. demonstrated that women with HCM did not have worse myopathy, but had a smaller LV cavity size and higher indexed maximum wall thickness [5]. Our data suggest that women with HCM had a higher LVM index, smaller LV cavity size and higher LVOT gradient, which might contribute to greater symptoms. In addition, we also found that female patients experienced worse LV remodeling than male patients after ASA, which might account for the poor prognosis for women. Myocardial fibrosis is known to increase in patients with HCM during LV remodeling and negatively influence the clinical course of HCM patients [14]. In this study, we found that female patients had a larger amount of LGE than men, which were both positively related to LV mass.

The mechanisms behind sex differences in HCM are currently not well understood. Sex differences in the hypertrophic response, fibrosis and gene expression suggest that physiological sex differences might play a role [15, 16]. Women are more likely to have concentric remodeling in response to pressure overload and, thus, have smaller LV diastolic chamber volumes, with higher EF [17]. Additionally, a higher degree of fibrosis in female myectomy samples was present than in male myectomy samples [15, 18], which was consistent with our study, indicating that the exacerbated LV remodeling in females might be caused by increased fibrosis, as replacement fibrosis is thought to be a potentiating element of LV remodeling in HCM [19].

Consistent with previous studies [7, 8], our study also found that ASA therapy significantly reduced LV mass and LVOT gradient in both men and women. There was a small increase in LV ejection as a result of a significant increase in LVEDV and a small but significant increase in LVESV at the 16-month follow-up. Previous studies have suggested that female sex is associated with worse survival after septal myectomy [20–22]. Furthermore, a recent study demonstrated that female patients with HCM undergoing ASA tended to have a poor prognosis compared with men in the Chinese population [23]. There is currently no clear explanation for why men fare better after ASA. In our study, men had more favorable reverse remodeling after ASA. Multiple regression analysis suggested that the main predictor of reverse remodeling for each category was the baseline level of the LVM index and change in the LVOT gradient, which might contribute to better survival in men. These findings were similar to Sorajja et al.’s result that the severity of the residual LVOT gradient after septal ablation is an important determinant of long-term outcome [24]. As LV remodeling might increase ventricular diastolic stiffening and lead to the compromise of diastolic reserve, which contributes to the greater rate of heart failure in women [5, 25], and LV mass proved more sensitive in predicting the outcome, it is reasonable to speculate that more LV mass regression was associated with greater improvement of diastolic function and, thus, with a better prognosis.

The underlying mechanisms for sex-based differences in the outcome after ASA of sarcomeric HCM are not well understood. However, the age of diagnosis or disease penetrance might be delayed, which might lead to a higher LVOT gradient and worse diastolic function abnormalities in women, thereby contributing to a higher incidence of HF and mortality in women with HCM [26]. Furthermore, women were more likely than men to demonstrate obstructive physiology and abnormalities of diastolic function, including greater pulmonary artery systolic pressure despite similar degrees of hypertrophy, even in HCM patients undergoing myectomy [2, 27]. In addition, this phenomenon might be related to the genetic and endocrine differences between males and females. Sex hormones might modulate the disease phenotype in HCM [28, 29]. Female sex was protective against disease manifestation and progression in a transgenic murine HCM model with myosin heavy-chain mutation [30] and troponin T mutation [31]. In our study, 90.6% of female patients were menopausal women and had worse survival than male patients, suggesting that postmenopausal endocrine changes might impact the clinical course in HCM [2, 32]. Moreover, women were more likely than men to have sarcomere pathogenic variants. Sarcomere variants that cause HCM have been shown to impair diastolic relaxation and lead to a greater burden of heart failure [26]. Other factors, such as societal and cultural factors, might influence the delayed referral and symptom assessment for women [21].

Previous studies have demonstrated that myocardial fibrosis detected by LGE is progressive in some HCM patients and that fibrosis progression is associated with adverse cardiac remodeling [33–35]. However, no studies have yet assessed the extent of LGE after ASA in long-term follow-up. Our study demonstrated that the extent of LGE did not significantly increase after ASA. Todiere et al. reported that the increase in LGE extent preferentially occurred in the apical pattern of LV hypertrophy [33]. However, myocardial hypertrophy was mainly involved in the interventricular septum and/or the anterior wall in the patients in our study. Second, Conte et al. and our study showed that the extent of LGE was significantly correlated with LV mass [36]. The favorable LV reverse remodeling might support the mechanistic explanation for the LGE without progression after ASA. Third, genotypic analysis for the screening of sarcomeric mutations was not performed in our study, and a genetic predisposition might condition the rate of progression of fibrosis [33, 35].

### Study limitations

There are some limitations in the study. First, the main limitation was the small size of the population. Moreover, due to higher disease penetrance in males, our study did not use a matched-control group design, and the females enrolled in our cohort were older but more symptomatic than the males. However, the baseline risk factors, including hypertension, diabetes and atrial fibrillation, were similar between the sexes. Moreover, in the multivariate analysis, variables, such as age as a dependent factor, were considered and had no effect on the final result. Second, the dropout rate was low for a CMR-based study, and the population was not consecutively recruited for the research, which might have led to the possible existence of selection bias. Third, although we provided LGE by CMR to measure fibrosis, we did not measure extracellular volume fraction (ECV) by T1 mapping. LGE is usually used to measure focal fibrosis, and T1 mapping might be used to quantify ECV as a marker of diffuse fibrosis [37]. Fourth, the precise mechanisms underlying sex-specific differences were not investigated in this study. Whether hormones, such as estrogen and androgen, are involved in the prognostic role of reverse LV remodeling as well as survival outcomes in patients with HCM needs further investigation by new studies.

## Conclusions

Our current results demonstrated that male patients with obstructive HCM had more favorable reverse remodeling with greater LV mass regression than females. Furthermore, a lower reduction in the LVOT gradient and a higher baseline LVM index were associated with poor LV remodeling and poor prognosis in women after ASA.

### Perspectives and significance

Hypertrophic cardiomyopathy (HCM) is an inherited disease characterized by marked genetic and prognostic heterogeneity. The relationship between sex and the clinical outcome after ASA in HCM remains incompletely resolved. In our study, we investigated whether sex differences affect the process of LV remodeling and outcome after ASA using CMR in obstructive HCM. Women with HCM presented at a more advanced age and with worse LV remodeling than men. After ASA, men experienced more favorable LV reverse remodeling than women. Women with HCM had worse relative composite endpoint than men. Sex and LV mass preablation were independent predictors of cardiovascular outcomes after multivariate analyses. Our findings indicated that sex appears to be a significant modifier in HCM patients receiving ASA treatment and highlighted the need for a different approach to women with HCM, such as improving women’s awareness of diagnosis and follow-up management as well as earlier referral for advanced therapies (e.g., septal reduction therapy and implantable cardioverter–defibrillator (ICD) implantation). Additionally, studies are needed to explore the influences of sex hormones on LV reverse remodeling as well as the clinical outcome after ASA in obstructive HCM in the future.

## Data Availability

The datasets during and/or analyzed during the current study are available from the corresponding author on reasonable request.
